# Professor Johannes V. Mikulicz-Radecki

**Published:** 1905-08

**Authors:** 


Professor Johannes v. Mikulicz-Radecki, died at Breslau, June 14, 1905, aged 55 years, of carcinoma of the stomach. Dr. Mikulicz was one of the most eminent surgeons of his day and in every way a lovely character. His visit to Buffalo two years ago, when he was entertained at dinner by a group of thirty physicians, will be remembered always by everyone present, and his tragic death will be regretted by his colleagues all over the world.

